# Comparative genomics of flowering behavior in *Cannabis sativa*

**DOI:** 10.3389/fpls.2023.1227898

**Published:** 2023-07-27

**Authors:** Laura Steel, Matthew Welling, Nic Ristevski, Kim Johnson, Anthony Gendall

**Affiliations:** Australian Research Council Research Hub for Medicinal Agriculture, La Trobe Institute for Sustainable Agriculture and Food, Department of Animal, Plant and Soil Sciences, School of Agriculture, Biomedicine and Environment, La Trobe University, Bundoora, VIC, Australia

**Keywords:** *Cannabis sativa*, flowering time, genomics, MADS-box, PEBP (phosphatidylethanolamine-binding)

## Abstract

*Cannabis sativa* L. is a phenotypically diverse and multi-use plant used in the production of fiber, seed, oils, and a class of specialized metabolites known as phytocannabinoids. The last decade has seen a rapid increase in the licit cultivation and processing of *C. sativa* for medical end-use. Medical morphotypes produce highly branched compact inflorescences which support a high density of glandular trichomes, specialized epidermal hair-like structures that are the site of phytocannabinoid biosynthesis and accumulation. While there is a focus on the regulation of phytocannabinoid pathways, the genetic determinants that govern flowering time and inflorescence structure in *C. sativa* are less well-defined but equally important. Understanding the molecular mechanisms that underly flowering behavior is key to maximizing phytocannabinoid production. The genetic basis of flowering regulation in *C. sativa* has been examined using genome-wide association studies, quantitative trait loci mapping and selection analysis, although the lack of a consistent reference genome has confounded attempts to directly compare candidate loci. Here we review the existing knowledge of flowering time control in *C. sativa*, and, using a common reference genome, we generate an integrated map. The co-location of known and putative flowering time loci within this resource will be essential to improve the understanding of *C. sativa* phenology.

## Introduction

*Cannabis sativa* L. is a monotypic, predominantly dioecious, annual herb of the Cannabaceae family (Small and Cronquist, 1976). Plants are diploid (2n = 20) with an estimated haploid genome of 818 Mb for females and 843 Mb for males (van Bakel et al., 2011; Divashuk et al., 2014; Small, 2015)*. C. sativa* has been cultivated in Eurasia for several thousand years and is now cultivated globally (Salentijn et al., 2015) due to its industrial (Karche, 2019), ornamental (Hesami et al., 2022b), nutritional (Krüger et al., 2022), medicinal, and recreational (Hesami et al., 2022a) applications. The genus *Cannabis* is widely accepted as comprising of a single species, *C. sativa* L. (Linnaeus), with highly polymorphic subspecies, *sativa*, *indica*, and *ruderalis* differing in phenotypic characteristics (Small and Cronquist, 1976; Sawler et al., 2015; Small, 2015; McPartland, 2018; Zhang et al., 2018a). For regulatory and agronomic purposes, *C. sativa* plants are classified based on the level of the phytocannabinoid intoxicant Δ9-tetrahydrocannabinol (Δ9-THC). Plants grown for industrial uses, such as those used for textiles and food, have a limited concentration of Δ9-THC. The level of Δ9-THC allowed in industrial-use plants can vary depending upon the jurisdiction but is typically between 0.2-1% (Salentijn et al., 2015). Plants containing less than 0.3% Δ9-THC in dried flower are generally classified and regulated as industrial hemp, with plants that exceed this threshold classified as drug-type (Hesami et al., 2020). Plants grown for fiber are typically taller and have less branching than drug-type plants grown for medicinal or recreational end-use (Salentijn et al., 2015). In contrast to industrially grown forms of *C. sativa*, drug-type plants are generally grown in controlled (indoor) environments, have compact inflorescences and exhibit greater stability in chemical profile (Upton et al., 2016). Biological activity of *C. sativa* is associated with the chemical constituents it produces, with phytocannabinoids such as cannabidiol (CBD) and Δ9-THC principally associated with medicinal effects (Beal et al., 1995; Devinsky et al., 2017).

Flowering is characterized by the transition from a shoot apical meristem to a floral meristem, which gives rise to a single flower or cluster of flowers, known as an inflorescence (Raghavan, 2000). An inflorescence is regarded as the reproductive part of the plant and can be comprised of the branches which bear the flowers and accessory structures (Prenner et al., 2009). The flowering process is a progressive sequence of physiological changes and developmental events, consisting of four key stages; floral initiation, floral organization, floral maturation, and anthesis [reviewed in (Raghavan, 2000)]. Floral initiation is characterized by the formation of floral primordia and marks the end of the vegetative phase. During floral organization, differentiation of individual floral parts takes place, with changes in the shoot apical meristem initiated by physiological and molecular changes in other parts of the plant (Chailakhyan, 1968). Floral maturation follows and this includes the formation of spore-producing tissues. The final stage is anthesis where flowers release pollen and styles have developed. The timing of flowering is essential to maximize reproductive success (Amasino, 2010), and the activation of floral meristem identity genes can be triggered by different pathways, including photoperiod-dependent, temperature-dependent (including vernalization), age-dependent (autonomous) and phytohormone-dependent (e.g., gibberellic acid (GA)) flowering pathways [reviewed in (Salentijn et al., 2019)]. For many plant species, flowering competency and responsiveness is contingent upon development from the juvenile to adult stage, even in the presence of inductive cues (Hyun et al., 2016). Interest in understanding the molecular components governing *C. sativa* flowering has accelerated over the last decade as jurisdictions amend legislation which constrained commercial production and scientific research (Nahtigal et al., 2016). Despite these developments, *C. sativa* remains an under-researched crop, with the genetic mechanisms governing its flowering pathways still largely undefined.

Here we examine the current knowledge of flowering time control in *C. sativa* and combine data from multiple sources using a common reference genome. This comparison of data from several quantitative trait loci (QTL) analyses and genome-wide association studies (GWAS) highlights key regions of the genome that contain putative regulators of flowering that have not yet been linked to flowering behavior in *C. sativa*. The current models for flowering time control are also described in the context of *C. sativa* flowering behavior and putative candidate flowering time genes are functionally classified by comparative analysis with known flowering time gene families.

## Materials and methods

### *C. sativa* growth conditions

**Figure 1:**
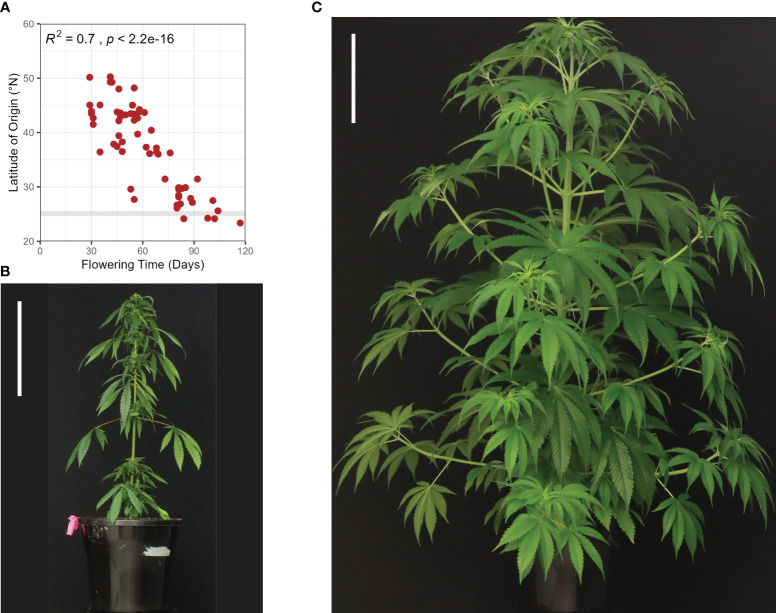
Variation in flowering and phenotypic characteristics of female hemp plants (*Cannabis sativa* L.): **(A)** Cannabis flowering time displays a strong latitudinal gradient for genotypes grown in a uniform environment The horizontal grey line indicates the latitude at which flowering time of different *C. sativa* varieties (indicated by the red dots) was assessed (25°N) under field conditions in natural short-day (SD) conditions (12-13 hours of daylight). Data adapted from Chen et al. (2022) and Zhang et al. (2018a). Photoperiod-insensitive (Autoflowering) cultivar Katani **(B)** and photoperiod-responsive cultivar Bama 4 **(C)** seven weeks post-sowing, after 40 days in SD, flower-inducing conditions. Scale bars are 23 cm.

All *C. sativa* plants were grown under an Authority for Low THC Cannabis, Authority Number 2019/01, issued by Agriculture Victoria. Plants were grown in controlled environment rooms at 24°C with 55% humidity using Philips metal halide lighting at ~415 µmol m^-2^s^-1^ (short-day) and ~150 µmol m^-2^s^-1^ (long-day). The plants used in **Figure 1** were grown from seeds, individually sown at a depth of 1.5 cm in soil media consisting of one-part perlite, one-part peat moss, and one-part vermiculite, with dolomite (1 g L^-1^). Seeds were sprayed with reverse osmosis (RO) water daily. Seedlings were transplanted into 500 ml pots 8 days post-sowing and then into 8 L pots at 31-33 days post-sowing. Seedlings were held in long-day (LD) conditions (18/6 h light/dark) for ~24 hours after transplant into 500ml pots, before transfer to short-day (SD) conditions (12/12 h light/dark). Plants were imaged after 40 days in SD conditions. Plants in LD conditions were watered daily using RO water supplemented with 0.4% (v/v) CANNA Classic Vega A and 0.4% (v/v) CANNA Classic Vega B. Plants in SD conditions were watered daily using 0.4% (v/v) Canna Classic Flores A and 0.4% (v/v) Canna Classic Flores B in RO water.

**Figure 2:**
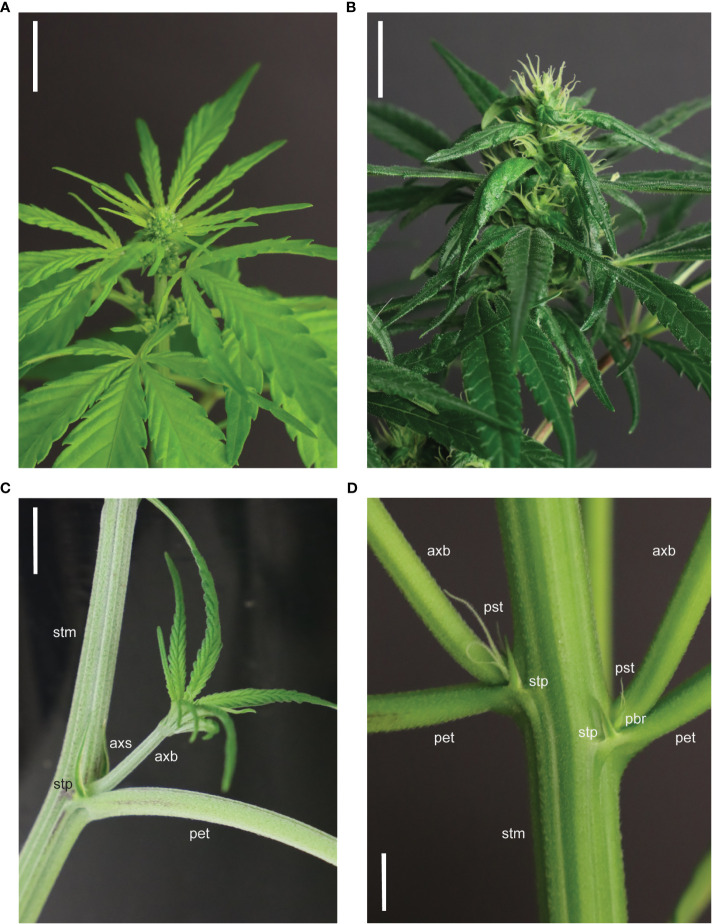
Terminal and solitary flowering phenotypes in *Cannabis sativa* L. **(A)** Staminate male flowers on an autoflowering *C. sativa* plant (Katani) in LD conditions (imaged 23 days post-sowing). **(B)** Pistillate female flowers on an autoflowering *C. sativa* plant (Katani) in SD conditions (imaged 51 days post-sowing, after 28 days in LD and 23 days in SD conditions). **(C)** Vegetative *C. sativa* anatomy at a basal node of a *C. sativa* plant (Bama) in LD conditions, depicting the axil of the stipule (axs), stipule (stp), axillary branch (axb), petiole (pet), and stem (stm). **(D)** Solitary flowers (stigma, style, perigonal bract and stipule) at the 6^th^ node of a *C. sativa* plant (Katani) flowering in LD conditions (imaged 37 days post-sowing), depicting the perigonal bract (pbr), stipules (stp), axillary branches (axb), petioles (pet), stem (stm), and pistils (pst; stigmas and style). Scale bars in **(A**, **B)** are 1 cm and scale bars in **(C, D)** are 2 cm.

**Figure 3:**
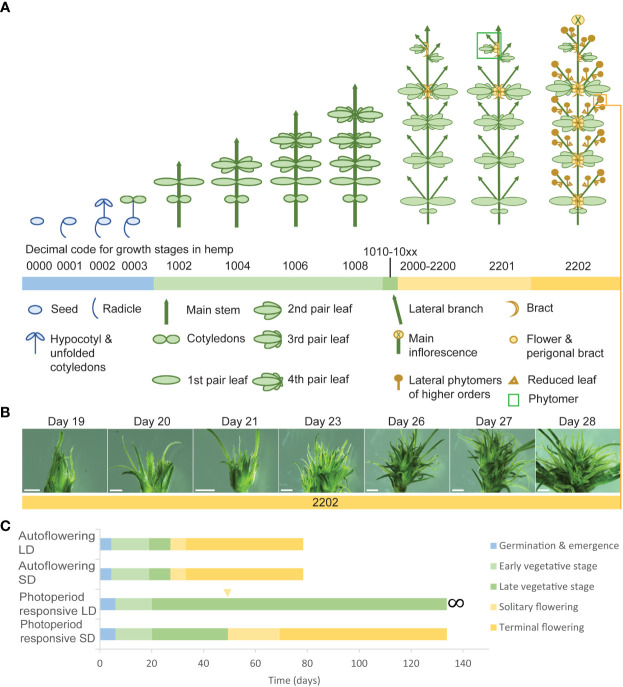
Development and flowering in *Cannabis sativa* L. **(A)** An illustration of select decimal growth stages in female hemp plants (*Cannabis sativa* L.), including germination and emergence (0000-0003), early vegetative stage (1002-1008), late vegetative stage (1010-10xx), and flowering (2000-2202) (Mediavilla et al., 1998). Schematic diagrams informed by Spitzer-Rimon et al. (2019). **(B)** Flower development in female hemp plants (*Cannabis sativa* L.) propagated from cuttings. Left to right: Day 19, 20, 21, 23, 26, 27, 28, where ‘Day’ is a measure of the number of days in a SD photoperiod. Each flower was sampled from an individual clone at the apex of an axillary branch at the third node, at the same time of day for the period 19-29 days after transfer to SD conditions (24°C and 55% relative humidity). Scale bars are 1 mm for Days 19 & 20 and 2 mm for Days 21-29. **(C)** Comparative time (days) spent in the three principal growth stages, germination and emergence, vegetative stage (early & late), and flowering (Mediavilla et al., 1998), for autoflowering and photoperiod-responsive female hemp plants in LD and SD conditions (24°C and 55% relative humidity). The flowering stage is divided into the time between solitary flower induction and terminal flower induction (solitary flowering) and the time between terminal flower induction and 95% seed maturity (terminal flowering). The yellow arrow indicates the point after which solitary flowers may form on photoperiod responsive plants in LD conditions.

Plants used in **Figure 2** were grown from seed, as described above (**Figures 2A, B, D**: *C. sativa* var. Katani), and a cutting (**Figure 2C**: *C. sativa* var. Bama 4) in LD conditions, as described below. Flowers used in **Figure 3** were sampled from clones from *C. sativa* var. Bama 4. The cuttings were rooted in GRODAN rockwool cubes using CLONEX purple rooting hormone and held vegetatively for 26 days under LD conditions. Five days before transfer to SD conditions, cuttings were transplanted into 1.15 L pots with soil media as described above. Flower samples were imaged using a Leica M80 dissecting microscope, fitted with a TL3000 Ergo light source.

### Mapping of GWAS and QTL markers

In a previous GWAS analysis, Petit et al., (Petit et al., 2020a; Petit et al., 2020b) (**Table 1**) mapped RADSeq markers to scaffolds of the ‘Purple Kush’ genome (canSat3, GCA_00230575.1). The Purple Kush genome is highly fragmented and consists of 12,836 scaffolds (van Bakel et al., 2011; Hurgobin et al., 2021). To improve on this approach and to unify data from different studies, we identified those scaffolds in the ‘Purple Kush’ genome with SNP markers significantly associated with flowering (LOD scores > 4) (Petit et al., 2020a) and aligned them to the cs10/CBDRx v2 reference genome (annotated from genotype CBDRx:18:580, GCF_900626175.2) using Minimap v2.17 (Li, 2018). Aligned regions containing markers were identified and plotted as separate tracks on a cs10 chromosome karyotype plot using Circos v 0.69-9 (Krzywinski et al., 2009), indicating the association statistic (LOD score) for flowering traits: ‘Beginning of flowering’, ‘Full flowering’, and ‘Length of vegetative phase’ scored at three distinct environments.

**Table 1:** Summary of flowering time studies in *Cannabis sativa*.

Study Type	Description	Reference Genome	Reference
BSA	*Autoflower1:* F_2_ plants derived from Otto II (late, photoperiod sensitive) x KG9202 (autoflowering). *Autoflower1:* Segregating in the ‘TJ CBG’ population. *Early1*: Segregating in the ‘Umpqua’ cultivar.	cs10/CBDRx v2 (GCF_900626175.2)	Toth et al., 2022
BSA	Three F_2_ populations, with one autoflowering parent, scored for flowering in Oregon USA.	Abacus (GCA_025232715.1).	Bakker et al., 2021
GWAS	RAD Seq using 123 hemp accessions, grown in three locations across Europe.	canSat3/PK (GCA_0002307575.1)	Petit et al., 2020a
QTL	372 F_2_ plants derived from a USO-31 (early/autoflowering) x Carmagnola (late) cross, grown in field in Colorado, USA.	Finola (GCA_003417725.2)	Woods et al., 2021
Gene Expression	RNASeq analysis of pre- and flowering-nodes in photoperiod-independent Volcani Line #213.	cs10/CBDRx v2 (GCF_900626175.2)	Spitzer-Rimon et al., 2022
Gene Expression	qRT-PCR expression of selected flowering-time genes in two wild and two cultivated varieties.	N.A.	Chen et al., 2022
Gene Expression	qRT-PCR expression of COL genes in four hemp varieties.	N.A.	Pan et al., 2021
Phenotyping	Genetically diverse female hemp plants crossed with ‘TJ’s CBD’ to generate 17 common families. Six families produced using two inbred S_1_ selections of ‘TJ’s CBD’.	N.A.	Carlson et al., 2021
GWAS Gene Expression	192 F_2_ plants (auto-flowering x photoperiod sensitive) grown indoors and genotyped using a SeqSNP chip with 5,000 custom markers RNA-Seq performed on samples taken from 54 F_2_ plants segregating for the auto-flowering trait grown under LD conditions	Purple Kush (ASM23057v4)	Leckie et al., 2023
Phenotyping	Controlled crosses using tetraploid parents of CBD-dominant cannabis photoperiod-sensitive cultivars Kentucky Sunshine, Wife, and Abacus (non-tetraploid) and autoflowering cultivars Purple Star, Tsunami, and Wilhelmina	N.A.	Kurtz et al., 2023
BSA	245 F_2_ plants resulting from ‘Felina 32’ × ‘FINOLA’ F_1_ offspring from four F_1_ female individuals (one male F_1_ pollen donor) grown under natural glasshouse conditions (long days, Dublin, Ireland, June-September 2020).	Finola (GCA_003417725.2)	Dowling et al., 2023

BSA, bulked segregant analysis; GWAS, genome wide association study; QTL, quantitative trait loci; N.A, not applicable.

A similar approach was used to map USO-31/Carmagnola QTLs described by Woods et al. (2021) to the cs10/CBDRx v2 reference genome (**Table 1**). Regions containing USO-31/Carmagnola polymorphic SNP markers positioned in the ‘Finola’ genome (GCA_003417725.2) were positioned in the cs10/CBRDx v2 using Minimap 2, as above, to define the endpoints and peaks of the four ‘Days to Maturity’ (DTM.1 through DTM.4) QTLs.

Genomic coordinates were extracted and plotted for the genes identified by Ren et al. (2021) as under selection using cs10/CBDRx2 reference genome protein accessions. Candidate flowering time gene protein sequences reported in the cs10/CBDRx v1 annotation (GCA_900626175.1) that did not correspond to the protein accessions in the cs10/CBDRx v2 annotation were translated and aligned to the cs10/CBDRx v2 genome. Four gene models: evm.model.01.2361 (*LD*), evm.model.04.2071 (*EMF1*), evm.TU.01.2503 (*FPF*) and evm.TU.08.543 (*FES1*) did not correspond to the reported putative flowering time genes and these were excluded.

### Flowering gene identification

Arabidopsis Gene Initiative (AGI) locus codes for 306 ‘flowering time’ and 72 ‘pending flowering time’ protein-encoding gene candidates from *Arabidopsis thaliana* were obtained from FLOR-ID (Bouché et al., 2016) (accessed on 19 September 2022). Corresponding protein sequences for these *A. thaliana* genes were obtained from The Arabidopsis Information Resource (TAIR; https://www.arabidopsis.org/). For microRNAs, nucleotide sequences were used. DIAMOND v0.9.24 (Buchfink et al., 2015) was used to compare these *A. thaliana* sequences to the proteome of *C. sativa* cs10/CBDRx v2 (GCF_900626175.2) and the best hits with greater than 90% identity were identified as likely orthologs. The longest isoform for each candidate was taken as the corresponding *C. sativa* cs10/CBDRx v2 ortholog. The microRNA nucleotide sequences for csa-miR156, 159a – b, and 172a – g miRNAs were retrieved *via* BLASTn analysis of the cs10/CBDRx v2 genome (Das et al., 2015).

To validate this flowering gene identification approach, and to identify additional homologs, we also conducted an Orthofinder analysis (Emms and Kelly, 2015) using the same *C. sativa* cs10/CBDRx v2 and *A. thaliana* predicted proteomes. The cs10/CBDRx v2 genome annotation was then further manually examined and additional putative flowering time genes with the keyword annotation ‘flowering’, ‘flower’, ‘time’, ‘circadian’, ‘day’, ‘clock’, and ‘vernalization’ were extracted. Genes were classified using the previously defined categories (Bouché et al., 2016). *C. sativa* genes with no clear ortholog in *A. thaliana* were assigned to the category of the most similar *A. thaliana* protein, based on the Orthogroup analysis using Orthofinder. Locations of the *C. sativa* flowering time genes in the *C. sativa* genome were plotted using Circos (Krzywinski et al., 2009).

### MADS gene phylogenetic analysis

As the annotation for cs10/CBDRx v2 MADS genes is incomplete, and to resolve the relationships between MADS-domain members, we identified all MADS genes in the cs10/CBDRx v2 genome. An initial search utilized three *A. thaliana* Type I MADS genes (AT1G01530, AT1G31630, AT5G49490) and three *A. thaliana* Type II MADS genes (AT1G24260, AT5G23260, AT5G60910) to represent each subgroup of the MADS box gene family (Gramzow and Theißen, 2013). The cs10/CBDRx v2 genome was searched using protein, translated nucleotide and nucleotide BLAST (blastp, tblastx and tblastn) analyses. Duplicate sequences were removed. All *C. sativa* MADS protein sequences (**Supplementary Tables S1**, **S2**) were aligned using Clustal Omega in Geneious Prime 2022.0 (Sievers et al., 2011) and tentatively assigned to clades. Any proteins not containing a complete MADS domain were excluded. An alignment was then generated using Clustal Omega v1.2.4 to assign the CsMADs proteins, including *A. thaliana* and *Vitis vinifera* predicted protein sequences (Gramzow and Theißen, 2013), to a clade. The best-fit amino acid substitution model (JTT+R10) was identified using IQ-Tree, and a Maximum Likelihood phylogenetic tree was generated using IQ-TREE 1.6 (Nguyen et al., 2015) (**Supplementary Figure S1**). A tree of only the Type II sequences was also generated using the aforementioned parameters. Phylogenetic trees were exported to iTOL for visualization (Letunic and Bork, 2007). Details of the accession numbers and clade assignment of *Cs*MADS genes are in **Supplementary Table S2**.

### PEBP gene phylogenetic analysis

To resolve the relationships between PEBPs-domain members, we identified all PEBP-encoding genes in the cs10/CBDRx v2 genome using tblastn and *A. thaliana* FT (AT1G65480), TFL (AT5G03840), MFT (AT1G18100) and BFT (AT5G62040) protein query sequences. The *C. sativa* PEBP family protein sequences were aligned with PEBP proteins from *A. thaliana*, tomato (*Solanum lycopersicon*; (Cao et al., 2016; Song et al., 2020)), and *Chrysanthemum seticuspe* ((Oda et al., 2011) using Clustal Omega v1.2.4 (Nguyen et al., 2015). Phylogenetic trees were exported to iTOL for visualization (Letunic and Bork, 2007). Full details of all protein accession numbers are in **Supplementary Table S2**. For clarity, here we have used the nomenclature suggested by Dowling et al. (2023)

### Analysis of protein-protein interactions

Protein sequences for 459 *C. sativa* cs10/CBDRx v2 flowering time gene candidates were imported into the Search Tool for the Retrieval of Interacting Genes/Proteins (STRING) database (v.11.5) (Szklarczyk et al., 2020) to generate protein-protein interaction networks. A short-list of 26 proteins from 6 categories of interest was generated using the following parameters: full STRING network, experiments and co-expression data, medium confidence (0.400) (**Supplementary Figure S2**).

### Expression analysis

*C. sativa* RNA-Seq datasets were retrieved from the European Nucleotide Archive Sequences were sourced from Braich et al. (2019); van Bakel et al. (2011) and Behr et al. (2016). Data from unpublished studies from the University of British Columbia (2020) and Michigan State University (2011) were also used. A full list of RNA-Seq data used in this study is available in **Supplementary Table S3**. RNA sequencing reads were checked for quality using FastQC (v0.11.9) and MultiQC (v1.12) (Andrews, 2010; Ewels et al., 2016). kallisto (v. 0.46.2) (Bray et al., 2016) was used for transcript abundance estimation and quantification based on pseudoalignment with the *C. sativa* cs10/CBDRx v2 reference. Sleuth (v. 0.30.0) (Pimentel et al., 2017) was used to quantify Transcripts per Million (TPM) for each gene. Sample replicates were averaged. Gene expression was visualized using pheatmap function in R, following a logarithm (log_2_(TPM+1)) transformation (Gu et al., 2016; Kolde, 2019).

## Flowering time regulation

### Diversity of flowering behavior in *C. sativa*

The photoperiodic induction of flowering (photoperiodism) can be used to classify plants as short-day (SD) plants, long-day (LD) plants and day-neutral plants. In SD plants, flowering occurs after periods of uninterrupted darkness, while in LD plants, flowering occurs in response to light periods longer than a certain critical length. *C. sativa* is considered a quantitative SD plant, with genotypes displaying a range of photoperiod thresholds for floral initiation (Amaducci et al., 2008a; Amaducci et al., 2012). Some genotypes have been reported to flower under 18 h of daylight (Chen et al., 2022), while most indoor commercially grown *C. sativa* plants require a 10-12 h uninterrupted dark period to induce flowering (Salentijn et al., 2019; Moher et al., 2021). Cannabinoid yields can be affected by lengthening the light period during flowering (Peterswald et al., 2023). THC producing lines, ‘Hindu Kush’ and ‘Northern Lights’, under a static 14 h light:10 h dark photoperiod showed a decline in THC concentration while plants from a CBD-producing line, ‘Cannatonic’, showed increases in CBD concentration (Peterswald et al., 2023). The time to visible floral induction under a short photoperiod can occur in as little as 1-2 weeks (Borthwick and Scully, 1954; Potter, 2014), with an increase in plant age at the time of transition reported to accelerate floral transition (Borthwick and Scully, 1954). Plants from the putative subspecific taxonomic grouping *C. sativa* var. *ruderalis* are reported to differ from the photoperiod-sensitive *C. sativa* var. *sativa* and *C. sativa* var. *indica* subspecies, with flowering induced in response to maturity (e.g., autoflowering) (Gloss, 2015). The vegetative-to-reproductive phase transition is indicated by the development of *de novo* solitary flowers and is thought to be regulated by internal signals (Spitzer-Rimon et al., 2019; Spitzer-Rimon et al., 2022). *Ruderalis* type plants are termed ‘autoflowering’, owing to their day-neutral flowering behavior, and these genotypes are thought to be responsible for the ‘autoflower’ trait in *C. sativa* populations (Gloss, 2015). It has been proposed that this trait follows a recessive, Mendelian pattern of inheritance, however, there is limited peer-reviewed research on this topic (Green, 2015; Toth et al., 2022; Kurtz et al., 2023; Leckie et al., 2023).

Adaptation to latitude appears to have contributed to changes in growth habit and sensitivity to photoperiodic induction. Plants can be classified into three genotypically distinct flowering time groups; early, intermediate, and late flowering. Early flowering genotypes grown for industrial end-uses can flower 40-60 days after sowing, intermediate after 60-90 days, and late after 90-120 days (Zatta et al., 2012). Early and intermediate genotypes are reported to have been bred at northern latitudes, with short growing seasons and long summer daylengths (**Figure 1A**). Cultivars adapted to higher latitude conditions flower earlier in lower latitudes where days are shorter, this can result in reduced biomass due to shortened growth duration (Amaducci et al., 2008b; Guo et al., 2013). Conversely, cultivars bred at low latitude are reported to have increased fiber yields when cultivated at higher latitudes (Guo et al., 2013), where the long vegetative growth, resulting from late flowering time, leads to greater stem biomass production. Our analysis of data from Chen et al. (2022) and Zhang et al. (2018a) comparing latitude of origin and flowering time (days) of genotypes grown in a uniform environment shows a strong negative correlation which supports the notion that plants bred at higher latitudes exhibit earlier flowering behavior (**Figure 1A**). We also flowered two industrial hemp genotypes in a 12 h light 12 h dark photoperiod under controlled environment conditions to highlight differences in plant morphology and flowering behavior (**Figures 1B, C**). The genotype bred at a higher latitude (**Figure 1B**; *C. sativa* var. Katani, Canada) exhibited earlier flowering behavior and reductions in orders of branching, plant height and biomass. In comparison, the lower latitude genotype (**Figure 1C**; *C. sativa* var. Bama 4, China) flowered later, with greater orders of branching, increased plant height, and biomass.

### Floral morphology and inflorescence structure

Sexual dimorphism is an important characteristic which has consequences for yield and the chemical composition of *C. sativa* plants (Welling et al., 2021). *C. sativa* has nine pairs of homomorphic autosomal chromosomes and a pair of heteromorphic sex chromosomes. Plants are usually diecious with distinct male and female plants (**Figures 2A, B**), however, plasticity in sexual phenotype can lead to hermaphrodite plants, also known as monecious phenotypes (Moliterni et al., 2004). Male plants (XY) typically flower earlier than female plants (XX) (Bócsa and Karus, 1998; Struik et al., 2000), possibly indicating that there are genes on the Y chromosome that accelerate flowering and/or repressors of flowering on the X chromosome, or that flowering time may be regulated by plant hormones involved in sex differentiation, such as gibberellic acid or ethylene (Galoch, 1978). Male plants produce pollen in hanging inflorescences and female plants produce pistillate flowers in dense clusters, separated by leafy bracts, while the morphology of monecious plants resembles that of female plants prior to the production of male flowers (Moliterni et al., 2004). Monoecious hemp accessions can be classified at flowering by their ratio of developed male to female flowers, which varies by cultivar and environment (Sengbusch, 1952; Faux et al., 2014). In addition to producing separate male and female flowers on a single plant, *C. sativa* can also produce bisexual flowers (Moliterni et al., 2004). The transition of *C. sativa* plants from vegetative growth to flowering can be indicated by the formation of undifferentiated primordia in the axils of stipules (protective structures, adjacent to the axillary buds (Heslop-Harrison and Heslop-Harrison, 1969) (**Figures 2C, D**), and, in some instances, by change of phyllotaxis from opposite to alternate (Bócsa and Karus, 1998) (**Figure 3A**; Stage 2000). After the appearance of floral primordia, dioecious male plants will form staminate flowers while female plants will develop bracts with no styles, which signifies the development of female flowers (Mediavilla et al., 1998) (**Figure 3A**; Stage 2200).

Defining the transition from vegetative to inflorescence flowering in *C. sativa* is complicated by the appearance of solitary flowers (**Figure 2D**). While a long photoperiod is considered ‘non-inductive’ for *C. sativa* plants, the development of solitary flowers in shoot internodes demonstrates that these plants are not strictly vegetative (Spitzer-Rimon et al., 2019). For *C. sativa* plants grown under a long photoperiod, differentiation of the first solitary flowers at the fourth to sixth internodes can occur (Cervantes, 2006). The induction of these solitary flowers is thought to be age-dependent and controlled by internal signals, as opposed to photoperiod (Spitzer-Rimon et al., 2019). Spitzer-Rimon et al. (2022) observed changes in the transcriptomic profile of flowering-related genes among nodes 4, 6, and 7 in female *C. sativa* seedlings grown under LD conditions. Flowering inducers (such as *MOTHER OF FT* (*MFT*), *SUPPRESSOR OF OVEREXPRESSION OF CONSTANS 1* (*SOC1*), *LEAFY* (*LFY*), and *APETALA1* (*AP1*)) were upregulated while flowering repressors (such as *TEMPRANILLO (TEM*), *TERMINAL FLOWER1 (TFL1*), and *BROTHER OF FT AND TFL1* (*BFT*) were downregulated and age-related orthologs (such as *SQUAMOSA-PROMOTER BINDING PROTEIN-LIKE* (*SPL*)s, see below) were activated in *C. sativa*. Given that solitary flowers can develop under both long and short photoperiods, it has been proposed that *C. sativa* is day-neutral in this aspect of flower-induction (Spitzer-Rimon et al., 2019). Further research directly comparing the timing of induction of solitary flowers in *Cannabis* plants grown under short and long days is required to determine whether the appearance of solitary flowers is photoperiod independent. It is still unclear as to whether the induction of solitary flowers signifies the end of the vegetative phase, as vegetative growth can continue at the SAM for the period between emergence of solitary flowers and terminal flowering at the shoot apex (**Figure 3A**; Stages 2201 - 2202).

Inflorescence flowering is marked by changes in the architecture of the shoot apex, which forms a highly branched compound raceme consisting of condensed branchlets and repeating phytomer structures (**Figures 2B**, **3A**; Stage 2202). These phytomer structures consist of an internode, foliage leaf (supported by a petiole), bracts, and solitary flowers (stigma, style, perigonal bract and stipule) (**Figures 2D**, **3A**; Stage 2201) (Spitzer-Rimon et al., 2019). Proliferation of these phytomer structures leads to the development of floral buds (**Figure 3B**), the main cultivation product of medicinal cannabis (Chandra et al., 2017). The compact nature of inflorescences can vary between genotypes and is affected by environmental stimuli, including light spectrum and intensity (Spitzer-Rimon et al., 2019; Danziger and Bernstein, 2021). While *C. sativa* is considered a short-day plant, some varieties exhibit photoperiod-independent flowering behavior (**Figure 3C**; ‘autoflowering’), producing flowers in response to maturity (Gloss, 2015). Similarly, not all plants will form terminal flowers at the apical meristem, even after several months of inflorescence flowering under inductive SD conditions (Spitzer-Rimon et al., 2019). These inconsistencies in flowering behavior indicate that the molecular mechanisms underlying floral initiation and inflorescence structure have a high level of heterogeneity in *C. sativa*.

**Figure 4:**
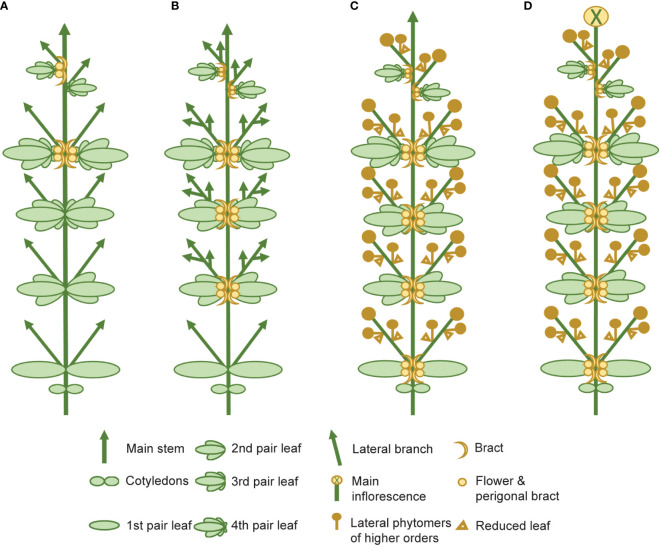
Schematic representing the four main stages of florogenesis proposed for use in flowering measurements of *Cannabis sativa* L. The four main events of florogenesis in female *C. sativa* plants, including **(A)** the induction of solitary flowers, signified by the development of the first bracts with stigma/style tissues, typically in the axils of the stipules, adjacent to the axillary buds **(B)** transition to higher order branching and proliferation of nodes at the shoot apex, **(C)** the onset of inflorescence flowering, marked by the formation of flower clusters, at the shoot apex and axillary branches, consisting of two or more bracts with pairs of stigmata (receptive to pollen), and **(D)** terminal flowering marked by the differentiation of the apical meristem by a terminal flower.

The complexity of the morphophysiological characteristics associated with flowering behavior in *C. sativa* has led to inconsistencies in nomenclature and in the reporting of these traits (Spitzer-Rimon et al., 2019; Petit et al., 2020b; Woods et al., 2021) (**Supplementary Table S4**). We propose that there are four main events which take place during florogenesis: 1) induction of solitary flowers, typically in the axils of the stipules (**Figures 2D**, **4A**), 2) formation of axillary branches and the transition to higher order branching (**Figure 4B**), 3) the onset of inflorescence flowering, marked by the formation of flower clusters at the shoot apex and axillary branches (**Figure 4C**), and finally 4) terminal flowering, when the apical meristem has transitioned to a terminal flower (**Figure 4D**). Changes in shoot apex architecture and inflorescence flowering can be inducible under short photoperiods and these characteristics appear to be regulated independently of solitary flower formation (Spitzer-Rimon et al., 2019).

### Flowering time and phytocannabinoid production

Phytocannabinoid content and yield is known to be highly variable and dependent upon genotype, growth stage, flowering behavior, and cultivation environment. Female *C. sativa* inflorescences are a rich source of hundreds of specialized metabolites, including phytocannabinoids (Welling et al., 2021; Welling et al., 2022). Phytocannabinoid biosynthesis is concentrated within glandular trichomes (Livingston et al., 2020), present on the perigonal bracts as well as other modified floral leaves within pistillate inflorescences. The capitate stalked trichome is the most abundant trichome morphotype in pistillate inflorescences and these and are principally responsible for the high concentration of phytocannabinoids in *C. sativa* plants (Livingston et al., 2020).

Many factors are capable of determining phytocannabinoid yield, including plant variety and age, planting density, and light intensity (Backer et al., 2019). Flowering time has a strong effect on phytocannabinoid accumulation, with rapid accumulation occurring in the first 3 weeks of inflorescence flowering (Stack et al., 2021). Importantly, both plant architecture and the accumulation of inflorescence biomass are strongly affected by flowering time (Stack et al., 2021). Comparisons between early and late flowering genotypes also indicate a limited trade-off between floral biomass and phytocannabinoid concentration, with genotypes producing the highest amounts of floral biomass also having the highest phytocannabinoid levels (Stack et al., 2021). This data indicates that the genetic manipulation of flowering pathways could be used as a viable strategy to increase phytocannabinoid yield within *C. sativa* commercial production systems.

### Inheritance of flowering traits

Whilst flowering traits in *C. sativa* appear to be quantitative and so reliant on the actions of many genes, early flowering time and autoflowering phenotypes appear to follow Mendelian expectations consistent with monogenic or multigenic modes of inheritance. A large range of variation in flowering behavior within and between cultivars, suggests multiple major effect loci contribute to this trait in *C. sativa* (Carlson et al., 2021; Stack et al., 2021; Toth et al., 2022), although segregation ratios for flowering time in ‘Umpqua,’ ‘Deschutes’ (~1:1 ratio of early- to late-flowering) and ‘Rogue’ (~1:3) populations suggests that a single locus is responsible for early flowering time (Stack et al., 2021). In seven *C. sativa* families segregating for early, mid, and late terminal flowering day, Carlson et al. (2021) observed that earlier flowering individuals were far less variable than those flowering later, suggesting a lower sensitivity to environmental cues. Segregation of S_2_ families indicated that with-in family variation in days to flower was the result of a common heterozygous parent for at least one major effect flowering time gene. Segregation was not indicative of a simple recessive trait, with the absence of a clear 3 late:1 early ratio in S_1_ progeny. Ratios were either ~1 late:1 early, ~2 late:1 early, all-early, or all-late, with a mean difference of ~10 days between the terminal flowering of early and late groups. This suggests that more than one gene is responsible for early flowering across these populations, although the limited sample size of these populations complicates the interpretation of inheritance patterns. In a separate population of the cultivar ‘Umpqua’, a major-effect flowering time locus, *Early1*, was also identified (spanning three significant peaks on Chr 1) (Toth et al., 2022). Bulked segregant analysis (BSA) indicated clear statistical significance for the *Early1* locus on cs10/CBDRx v2 Chr 1, with *Casein kinease-1 like protein 1* (LOC115705415) the strongest *Early1* candidate, although another 44 genes were also present across three confidence intervals linked to the early flowering phenotype.

The inheritance of photoperiod insensitivity appears less ambiguous than that of flowering time behavior. Toth et al. (2022) demonstrated that hemp photoperiod insensitivity (or ‘autoflowering’) is a recessive Mendelian trait (1:2:1). The *Autoflower1* locus was mapped to cs10/CBDRx v2 Chr 1 (17.74-22.94 Mb) (Toth et al., 2022) (**Table 1**). Heterozygous *Autoflower1* individuals were intermediate for flowering date and homozygotes exhibited earlier flowering behavior (Toth et al., 2022). This is consistent with the segregation of the autoflower trait in other F_2_ populations (Leckie et al., 2023), with several lines of investigation supporting the involvement of mutations in a *PSEUDO-RESPONSE REGULATOR 37* (*CsPRR37*) gene (Leckie et al., 2023). Gene dosage and incomplete dominance of the A allele at the autoflowering locus has also been reported among diploid and triploid genotypes (Kurtz et al., 2023), providing further evidence that photoperiod insensitivity is controlled by a single locus and is a homozygous recessive trait.

Despite recent advancements in the inheritance of flowering behavior, BSA, which compares a limited number of individuals within a segregating population and has been used extensively in *C. sativa* genomic analyses, can lack the statistical power to identify small effect QTL due to lower rates of observable recombination (Laverty et al., 2019). Moreover, many of these experiments have been conducted across heterogeneous environments using diecious parents with varying levels of heterozygosity (Toth et al., 2022; Kurtz et al., 2023; Leckie et al., 2023). As such, further research which makes use of more controlled environments, to delineate genetic contributions more accurately, as well as alternative breeding schemes are required to better understand the genetic basis underlying flowering behavior in natural populations of *C. sativa*.

## Genetic analyses of flowering in *C. sativa*

**Figure 5:**
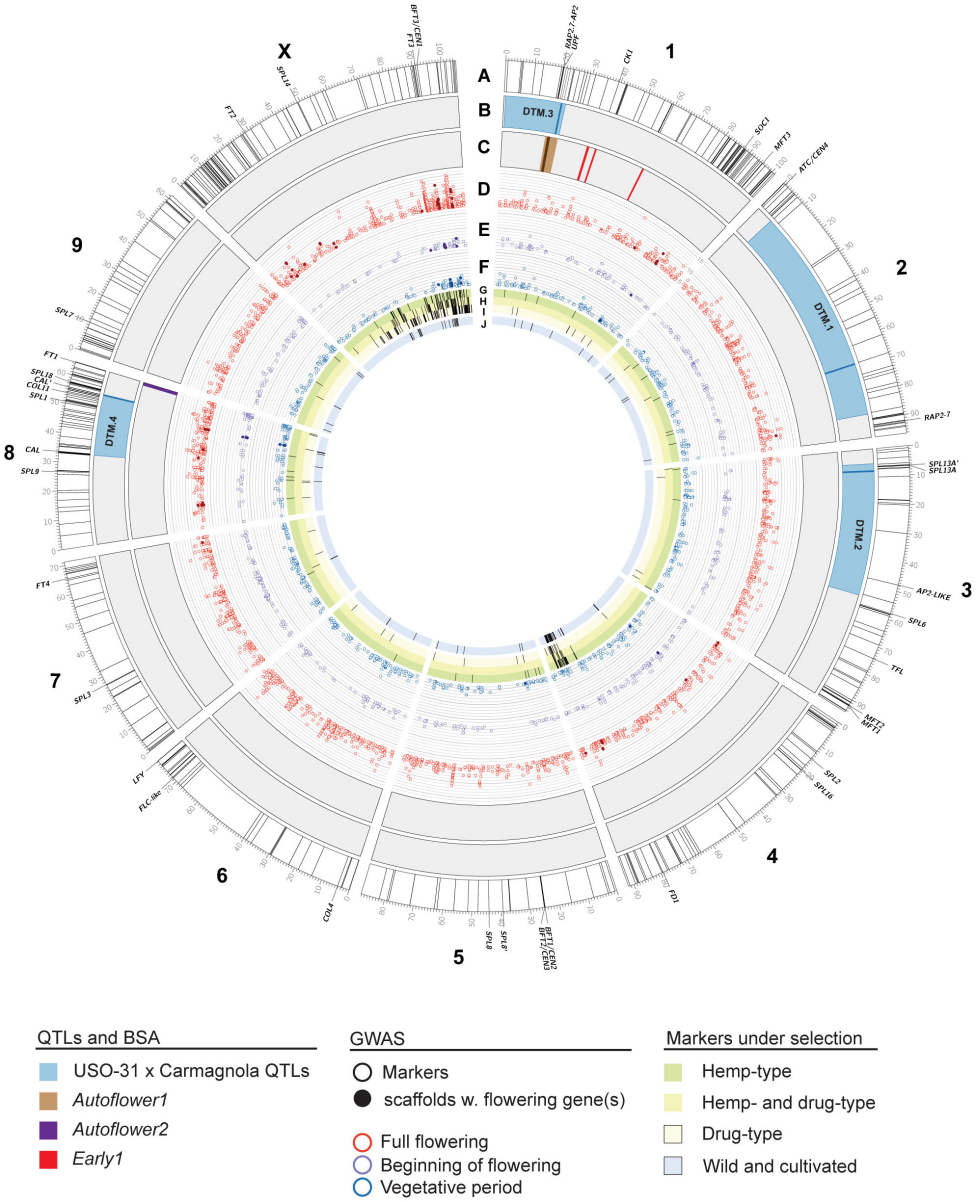
Genomics of flowering time in *Cannabis* sativa L. Chromosomes of the cs10/CBDRx reference genome (genotype cs10/CBDRx:18:580, GCF_900626175.2) are shown. Labelled genes are those that have been characterized or discussed in the text. **(A)** Locations of putative flowering time genes in the cs10/CBDRx genome are indicated with radial lines (**Supplementary Table S1**). **(B)** Locations of Days to Maturity (DTM) QTLs in the Carmagnola x USO-31 F_2_ population (Woods et al., 2021). Regions with LOD >1.5 are shaded blue, with peaks as solid lines. **(C)** Locations of markers associated with the *Autoflower1* (brown, with peak as a solid line), *Autoflower2* (purple) and *Early1* (red) loci (Toth et al., 2022, []Dowling et al., 2023). **(D**–**F)** GWAS markers associated (LOD >4.0) with full flowering **(D)**, beginning of flowering **(E)**, and the length of the vegetative period **(F)**. Solid symbols are scaffolds with flowering genes identified by Petit et al. (2020a). The scale is LOD 4 – 16. **(G**–**I)** GWAS markers under selection in hemp-type **(G)**, both hemp- and drug-type **(H)**, and drug-type **(I)** *C. sativa* strains (Ren et al., 2021). **(J)** GWAS markers under selection in wild and cultivated *C. sativa* strains (Chen et al., 2022).

Genomics has been pivotal to our understanding of the molecular mechanisms underlying flowering behavior in the model species *A. thaliana* and other important crop species. However, international narcotics conventions and associated legislation have constrained these analyses in *C. sativa* (Welling et al., 2016; Hurgobin et al., 2021), with the genetics of flowering time control only recently being reported in *C. sativa*. To date, ten studies have examined the genetic basis of flowering time (**Table 1**). These have used *C. sativa* genome assemblies of varying quality, completeness, and contiguity. This complicates comparative analyses between datasets and the identification of syntenic relationships between genomic intervals of interest. To facilitate comparison of these legacy studies, we generated a unified *C. sativa* CBDRx genome of flowering time genes. Regions of interest were mapped to a chromosome-scale reference genome of *C. sativa* to identify co-located QTL and genetic markers linked to flowering behavior, with intervals annotated by sequence similarity to known flowering time genes (**Figure 5**) (See Materials and Methods).

Several QTLs involved in flowering and sex determination have previously been identified by a genome-wide association study (GWAS)-based approach (Petit et al., 2020a), however, this analysis used a highly fragmented reference genome consisting of over ~135K unplaced scaffolds (**Table 1**). Despite this limitation, genes associated with light perception and transduction were identified in the QTL for ‘full flowering’. Our comparative genomic analysis aligned several regions containing genes associated with flowering time to the *C. sativa* cs10/CBDRx genome (**Figure 5**) and these were most commonly enriched for the GO term ‘Photoperiodism, light perception and signaling’ (**Table 2**, **Supplementary Table S1**). We identified 4 co-localized QTL regions on Chr X, 3, 8 and 1. Of particular interest are a cluster of genes on Chr X at c. 85-100 Mb, which do not coincide with described QTLs, but overlap with the Petit et al. (2020a) QTL for ‘full flowering’. This region includes two phosphatidylethanolamine-binding (PEBP) members, *CEN1* and *FT3* (**Figure 5**), that encode proteins involved in flowering time, and may represent a sex-dependent locus.

**Table 2:** FLOweRing Interactive Database (Flor-ID) descriptions for *Cannabis sativa* putative flowering time genes.

Flor-ID Keyword	Instances of keyword association with a gene
Aging	28
Ambient temperature	7
Circadian Clock	49
Flower development and meristem identity	40
Flowering time integrator	32
General	159
Gibberellins	19
Hormones	73
Photoperiodism, light perception and signalling	165
Response to cold	1
Sugar	9
Vernalization	40

Woods et al. (2021) produced an F_2_ population of 372 plants by crossing phenotypically distinct hemp cultivars, Carmagnola and USO31 (**Table 1**). Whole-genome sequencing of the F_2_ population (n = 372) using a legacy Finola genome identified four QTLs associated with days to maturity (DTM) (**Figure 5**). The corresponding locations for these QTLs in the cs10/CBDRx genome are Chr 1 (5.97- 23.04 Mb), Chr 2 (6.46 – 7.62 Mb), Chr 3 5.5 - 54.745 Mb) and Chr 8 (33.11 - 55.84 Mb (**Figure 5**). Interestingly, DTM.3 coincides with the location of *Autoflower1*, associated with early and photoperiod-insensitive flowering (Toth et al., 2022) (**Figure 5**). DTM.2 contains a pair of *SPL*s close to the peak at c. 8 Mb on Chr 3 (**Figure 5**; *SPL13A* and *SPL13A’*). *SPL* genes encode transcription factors (TFs) that promote *SOC1* expression, resulting in the activation of the floral meristem identity gene *LEAFY* in *A. thaliana* (Liu et al., 2007). Genes coding for TFs involved in the autonomous flowering pathway, including *SOC1* and *SQUAMOSA*, were also identified in Petit et al. (2020a) flowering time QTLs. DTM.4 (Chr 8, c. 25 – 60 Mb) is coincident with several flowering time candidates, including *COL11* and *SPL1* (**Figure 5**). *CsCOL11* demonstrates higher expression levels in early flowering varieties under SD conditions, while *CsSPL1* is upregulated during plant maturation, from node 4 to node 7, and believed to be involved in the vegetative to reproductive phase transition (Pan et al., 2021; Spitzer-Rimon et al., 2022).

**Figure 6:**
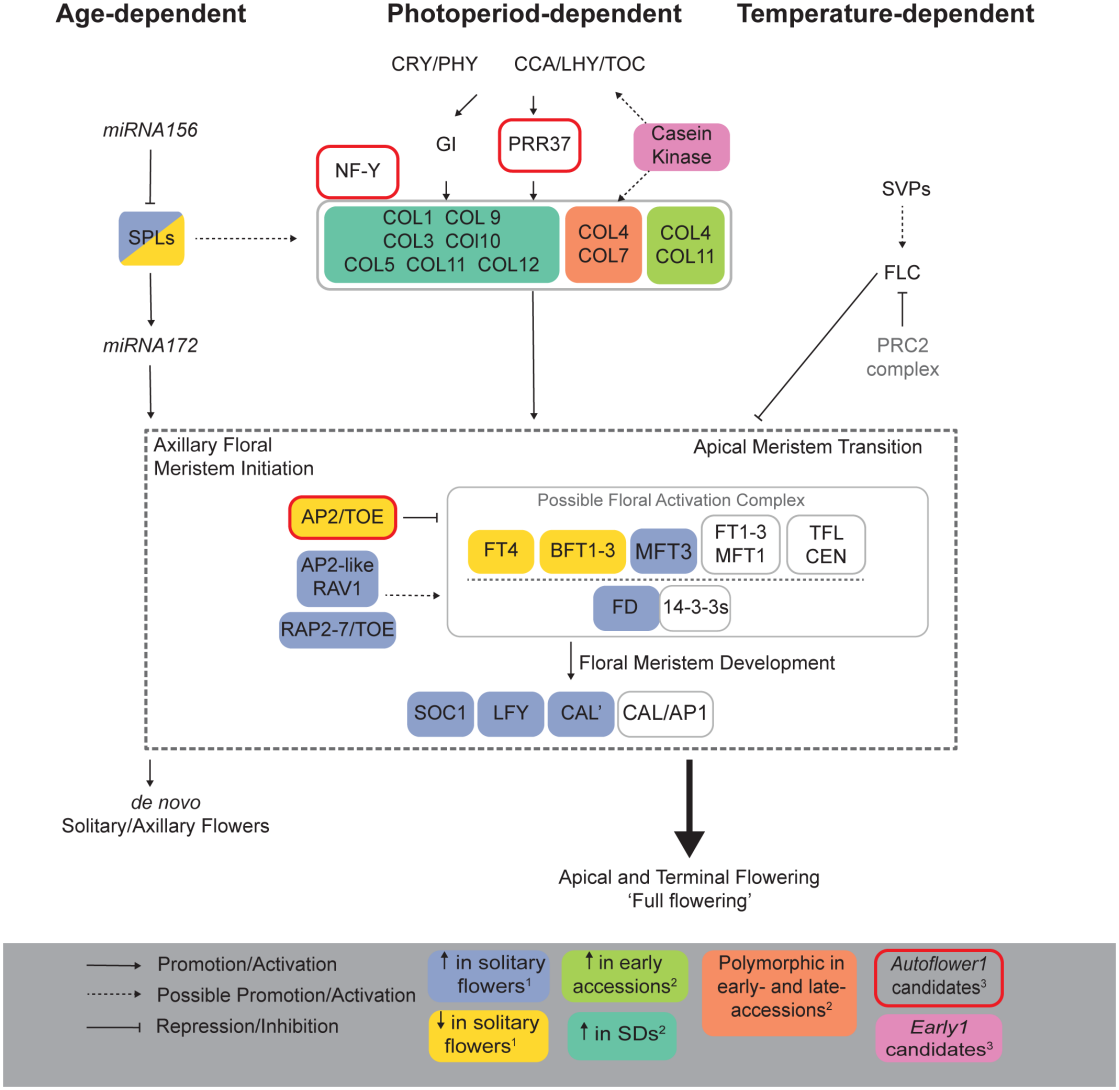
Putative flowering time pathways in *Cannabis sativa* L. Potential age-, photoperiod- and temperature-dependent pathways regulating flowering in *C. sativa* based on known pathways in model species (*A. thaliana*, soybean, and rice) as well as recently identified QTLs in hemp (Petit et al., 2020a). Superscripts indicate references: ^1^ Spitzer-Rimon et al. (2022), ^2^ Pan et al. (2021) and ^3^ Toth et al. (2022).

We also analyzed *C. sativa* cs10/CBDRx protein-encoding flowering time gene candidates to examine putative interaction networks. Analysis revealed groups involved in flower development and initiation and maintenance of inflorescence meristem identity, including 14-3-3 proteins, MADS (MCM1, AG, DEFA, and SRF-box) proteins, and PEBPs (**Supplementary Figure S2**). FD is a basic-leucine zipper (bZIP) transcription factor family protein responsible for positive regulation of flowering in *A. thaliana* (Abe et al., 2005). PEBPs TFL1, BFT and ARABIDOPSIS THALIANA CENTRORADIALIS (ATC) were present and are suggested to interact with FD (Hanano and Goto, 2011; Huang et al., 2012; Ryu et al., 2014). In *A. thaliana*, *ATC* and *TFL1* encode similar proteins, with TFL1 required to maintain an indeterminate inflorescence by preventing the expression of *AP1* and *LFY* (Conti and Bradley, 2007). FD interacts with FLOWERING LOCUS T (FT) to promote flowering, as FT activates the transcription of several floral meristem identity genes and is thought to act in parallel with LFY to induce flowering by regulating AP1 (**Figure 6**). Comparative genomic analysis indicated the presence of an *FD-like* gene at ~ 80 Mb on Chr 4 (**Figure 5**).

To determine if any of these regions were under selection, we also plotted data from Ren et al. (2021) and Chen et al. (2022) who examined selection and domestication in hemp and drug-types of cannabis. This revealed two regions coincident with several putative flowering time loci. One is located at ~85-90 Mb of Chr 4, close to the FD-like gene, while the other is a broader region encompassing much of the distal end of Chromosome X (~50-105 Mb), including FT3 and CEN1 (**Figures 5G–J**).

### Photoperiod-dependent pathways in *C. sativa*

**Figure 7:**
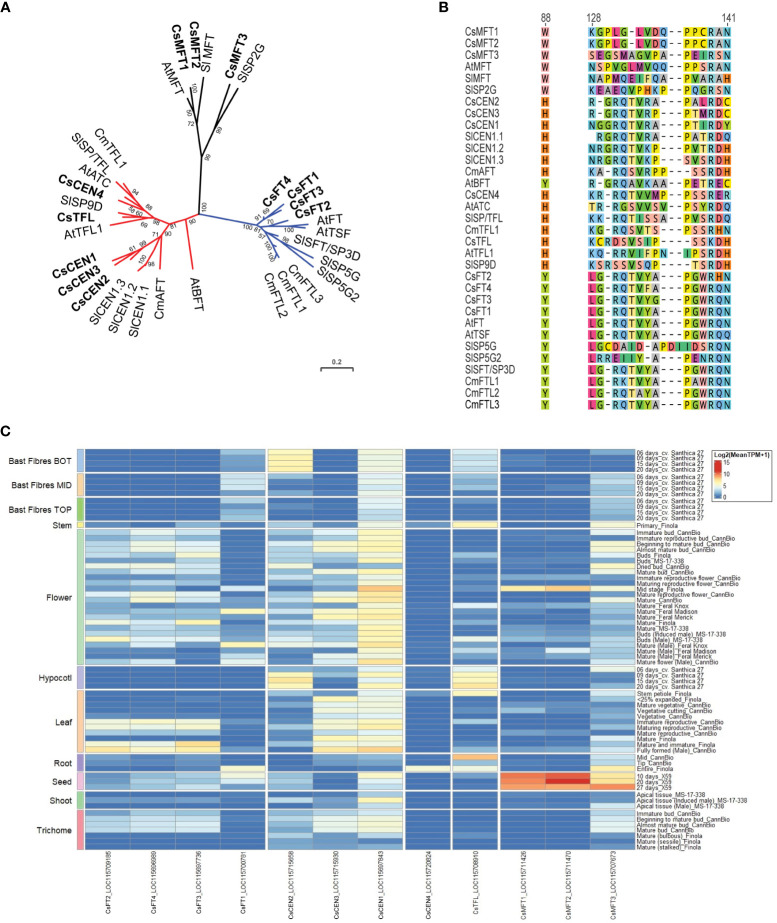
Phosphatidylethanolamine-binding protein family members in *Cannabis sativa* L. **(A)** Phylogenetic analysis of *C. sativa* PEBP proteins. Proteins were aligned using CLUSTAL in Geneious Prime, and a maximum-likelihood tree was produced using IQ-TREE with JTT+I+G4 parameters as the best model under AIC and BIC criteria (Minh et al., 2020). The tree was visualized with ITOL (https://itol.embl.de; (Letunic and Bork, 2007)). Numbers indicate percentage bootstrap support following 100 bootstraps (only values above 50 are shown). Species abbreviations: At – *A. thaliana*, Sl - *Solanum lycopersicon*, Cm – *Chrysanthemum seticuspe*. The scale is the average number of substitutions per site. **(B)** Alignment of the critical Y85/H88 and segment B regions of the PEBP proteins shown in **(A)**. Numbers correspond to amino acid residues in *A. thaliana FLOWERING LOCUS T* (AtFT). **(C)** Expression of *CsPEBP* family in different *C. sativa* tissues or stages of development. Details of RNASeq data sets are in **Supplementary Table S3**.

The photoperiod-dependent flowering pathway involves light-sensing proteins (phytochromes and cryptochromes) which coordinate with the circadian clock to regulate the expression of the phosphatidylethanolamine-binding protein (PEBP) family, including a sub-family related to the FT protein (**Figures 6A**, **7A**). PEBP members can function both as inducers and inhibitors of flowering. *C. sativa* is particularly sensitive to photoperiodic changes, with the time to flower reduced in SD conditions (Hall et al., 2012). The PEBP gene family is well represented in *C. sativa*, with both putative inducers and inhibitors of flowering present (see below) (**Figure 7A**). The flowering time network of the model species *A. thaliana* is well-defined with several pathways converging on floral integrator genes (Blümel et al., 2015), including *FT*, *TWIN SISTER OF FT* (*TSF*; (Amasino, 2010)), and *SOC1* (**Figure 7**). *FT* and its orthologs are synthesized in the leaves of several plant species and encode proteins that function as florigens and anti-florigens, promoting or inhibiting floral initiation at the shoot apex, respectively. *A. thaliana* possesses five phytochromes: PHYA through PHYE, the signals from which are received by the GIGANTEA-CONSTANS-FT (GI-CO-FT) signaling cascade. Stabilized by PHYA, the nuclear TF *CONSTANS* (*CO*) activates transcription of *FT* (Putterill et al., 1995; Samach et al., 2000). The *FT* locus produces florigen in the leaves which then travels to the shoot apical meristem to initiate flowering (Corbesier et al., 2007). *GI*, a circadian clock gene, facilitates the degradation of transcriptional repressors responsible for repressing the expression of *CO*, indirectly promoting *FT* (Sawa et al., 2007). *CO* indirectly upregulates the MADS-box TF gene *SOC1*, which activates the floral meristem identity gene *LEAFY* (*LFY*) to promote flowering (Yoo et al., 2005; Lee et al., 2008). *FLOWERING LOCUS C* (*FLC)*-*like* genes negatively regulate flowering time in the autonomous and vernalization flowering pathways, with elevated levels of *FLC* resulting in later flowering in *A. thaliana* (Sheldon et al., 2000). *FLOWERING LOCUS D* (*FLD*) codes for the FLD TF, which regulates *FLC*. FLD facilitates histone demethylation at the *FLC* locus, deactivating *FLC* expression and triggering flowering (He et al., 2003; Jiang et al., 2007). The overexpression of *TERMINAL FLOWER 1 (TFL1)/CENTRORADIALIS (CEN)-like* genes also delays flowering and alters flower architecture in *Hevea brasiliensis* (Bi et al., 2019) and CENTRORADIALIS (CEN)-like protein 1 (encoded by *CET1*) is highly expressed in the developing inflorescences of *A. thaliana* and *Antirrhinum* (Bradley et al., 1996; Bradley et al., 1997).

*CO-like* (*COL*) genes are TFs in pathways associated with growth and development, including the photoperiod-dependent flowering pathway (**Figure 6**). The *COL* gene family is known to regulate flowering under both SD and LD conditions, with negative regulators under both photoperiods in rice (*Oryza sativa;* a facultative SD plant), *OsCOL10*, *OsCOL13* and *OsCOL16* as well as *Hd1*, a promoter of SD dependent flowering that suppresses flowering under LD conditions (Yano et al., 2000; Sheng et al., 2016; Tan et al., 2016; Wu et al., 2017). Overexpression of *COL* genes in *A. thaliana* (*AtCOL3*, *AtCOL7* and *AtCOL8*) delays flowering while the overexpression of *AtCOL5* increases the expression of *FT* to promote flowering (Datta et al., 2006; Hassidim et al., 2009; Takase et al., 2011; Wang et al., 2013). Pan et al. (2021) conducted an analysis of the *CONSTANS-like* gene family in *C. sativa* (*CsCOL*) and identified 13 *CsCOL* genes (*CsCOL1 – CsCOL13*), unevenly distributed across 7 chromosomes and primarily located on Chr 10. Ten *CsCOL* genes were preferentially expressed in the leaves, two in the female flower (*CsCOL2* and *CsCOL3*), and one in the stem (*CsCOL13*). Most *CsCOL* genes identified by Pan et al. (2021) exhibited a diurnal oscillation pattern under SD and LD conditions and sequence analysis indicated amino acid differences for *CsCOL3* and *CsCOL7* among early flowering and late flowering varieties. At peak transcription levels, *CsCOL4* and *CsCOL11* expression levels were higher in the two early flowering varieties tested, compared to those of the two late flowering varieties. The reverse was true for *CsCOL6*, *CsCOL7*, *CsCOL9*, and *CsCOL12*. This indicates that there may be multiple *CsCOL* genes functioning as promoters or suppressers of flowering to regulate flowering time in *C. sativa*. While gene functions and mechanisms can differ between species, the apparent conservation of *GI*, *CO*, and *FT* in the flowering pathways of many crops (Watanabe et al., 2011), along with the photoperiod-dependent regulation of *FT-like* expression (Chen et al., 2022) and *COL* expression in *C. sativa* suggest that these may be ideal candidates in determining the regulation of flowering time in *C. sativa* and warrant further investigation.

In soybean (*Glycine max)*, a SD dicot, flowering time is regulated by *E* genes and *JUVENILE* (*J*), also known as *GmELF3* (Copley et al., 2018). *GmELF3* is orthologous to *A. thaliana EARLY FLOWERING3* (*ELF3*), that encodes a key component of the circadian clock (Lu et al., 2017). *E1* is a legume-specific TF and *E2*, *E3*, and *E4* are orthologous to genes associated with the regulation of flowering time in *A. thaliana*. *E2* (also *GmGIGANTEAa*) is an ortholog of *GIGANTEA* (*GI*), and *E3* (*GmPHYA3*) and *E4* (*GmPHYA2*) are orthologs of *PHYA*. Under long day conditions, GmPHYA3 and GmPHYA2 promote *E1* expression and inhibit *GmELF3* expression. E1 up-regulates *GmFT4a* and down-regulates *GmFT2a* and *GmFT5a*, all of which are *FT* homologs (Xia et al., 2012; Nan et al., 2014; Zhai et al., 2014; Samanfar et al., 2017). *GmGIa* (a *GI* homolog) delays flowering under LD conditions by inhibiting *GmFT2a* (Watanabe et al., 2011). The *E1* to *E4* loss-of-function alleles result in photoperiod insensitive flowering due to increased *FT* gene transcript levels (Xu et al., 2013). Under SD conditions, GmELF3 represses *E1*, releasing the *E1* suppression of the *GmFT* genes, promoting flowering (Xia et al., 2012; Lu et al., 2017). Flowering time variation in soybean is caused, in part, by natural variation in the *GmFT* gene family (Jiang et al., 2019). *C. sativa* has two *GI* (LOC115708742 and LOC115722652), three *ELF3* (LOC115703149, LOC115697482 and LOC115707722) and three *PHY* homologs (*PHYA*: LOC115719277, *PHYB:* LOC115721719, and *PHYE*: LOC115697533) and, as such, these genes may assist in understanding variation in sensitivity to photoperiod in *C. sativa* (**Supplementary Table S1**).

Photoperiod affects many aspects of plant development, including the initial elongation of flower stalks, flower initiation (Blümel et al., 2015), meristem termination, bud dormancy and branching. Overexpression of *FT* homologs induces very early flowering in eudicot plants, such as tomato (*Solanum lycopersicum*; Lifschitz et al., 2006), and monocot plants, such as rice (Izawa et al., 2002; Kojima et al., 2002). *FT* and *TSF* also promote lateral shoot development in *A. thaliana*, independently of their effect on floral initiation (Hiraoka et al., 2013). Additionally, *BRANCHED1/TEOSINTE BRANCHED1-LIKE 1* TF, a key negative regulator of branching in *A. thaliana*, can inhibit the function of both *FT* and *TSF* (Niwa et al., 2013). A similar mechanism exists in *C. sativa*, given that a short photoperiod promotes intense branching of the inflorescence (Spitzer-Rimon et al., 2019). Research in day-neutral tomato (*Solanum lycopersicum)* has explored the nature of the relationship between branching and flowering, with late-flowering mutants showing a greater propensity to revert to vegetative functioning in the inflorescence. It has been suggested that there are common mechanisms between the inhibition of vegetative growth in the shoot apical meristem and the number of lateral meristems initiated in the inflorescence (Périlleux et al., 2014). *FA* (*FALSIFLORA*) and *SINGLE FLOWER TRUSS* (*SFT*) are the tomato orthologs of the *A. thaliana LFY* and *FT* genes, respectively (Molinero-Rosales et al., 1999; Lifschitz et al., 2006). Mutants *fa* and *sft* exhibit leaf production in the inflorescence (Allen and Sussex, 1996; Molinero-Rosales et al., 1999; Molinero-Rosales et al., 2004) with additive late-flowering phenotypes, indicating that the genes act in parallel pathways (Molinero-Rosales et al., 2004; Thouet et al., 2012). Conversely, *FA* and *SFT* are floral promoters, with overexpression of either accelerating flowering (Lifschitz et al., 2006; MacAlister et al., 2012). The early flowering tomato mutant *terminating flower* (*tmf*) exhibits a reduction in the number of vegetative phytomers, like that of plants overexpressing *FA* or *SFT* (MacAlister et al., 2012). *TMF* acts upstream of *FA* and independently of *SFT* to maintain a vegetative shoot apical meristem. Both *FA* and *LFY* are floral meristem identity genes, expressed in leaf primordia before flowering with expression increasing with transition from a shoot apical meristem towards a flowering meristem (Molinero-Rosales et al., 1999; Thouet et al., 2012).

The FAC consists of FT, a 14-3-3 protein, and FD and plays a vital role in promoting flowering in tomato (Pnueli et al., 2001) (**Figure 6**). SFT interacts with a 14-3-3 protein, in tomato, facilitating the interaction with SELFPRUNING (SP; an ortholog of TERMINAL FLOWER1)-interacting G-BOX (SPGB) to form the FAC (Pnueli et al., 2001). Song et al. (2020) examined the interactions between FTL1, a tomato FT paralog, SPGB and three 14-3-3 isoforms and determined that FTL1 interacts with 14-3-3/2 to form the FAC, with SPGB regulating tomato flowering. Allelic variation in *SELF-PRUNING 5G* (*SP5G*), an *FT* paralog, reduces the LD response and contributes to the loss of day-length-sensitive flowering in tomato (Soyk et al., 2017; Zhang et al., 2018b). *FTL1* was induced by SD conditions, as opposed to LD conditions, with transcript levels indicating a strong diurnal oscillation (Song et al., 2020). *SFT* is a floral inducer but does not respond to day length (Molinero-Rosales et al., 2004; Lifschitz et al., 2006), acting downstream of *FTL1* to regulate SD dependent flowering. Disruption of both *SP5G* and *FTL1* function induces day-neutral flowering in tomato, by enhancing or reducing *SFT* expression under LD or SD conditions (Soyk et al., 2017; Song et al., 2020). *SFT* induces early flowering in tomato and is conserved in other species (Lifschitz and Eshed, 2006; Lifschitz et al., 2006). In *C. sativa*, there are nine 14-3-3 and two FD putative homologs present in *C. sativa*, suggesting the existence of similar pathways (**Supplementary Table S1**).

Analysis of the expression of *FLOWERING LOCUS T-like* (*FT-like/LOC115697736/FT3*) and *CET1*/*LOC115697843/CEN1* in *C. sativa* accessions from different latitudes shows that wild accessions flowered under both LD and SD conditions and that the cultivated plants only flowered in SDs. *FT-like* expression was significantly higher in the wild accessions under LD conditions and was positively correlated with the latitude of origin. Cultivated plants showed low *FT-like* expression under LD conditions, while *FT-like* expression was high and rapidly followed flowering in all accessions under SD conditions, suggesting that *FT-like* may promote flowering. The relatively unchanged expression of *CET1* across developmental stages has been interpreted by some authors as evidence that flowering behavior is not controlled by autonomous or vernalization pathways and that cultivated *C. sativa* has adapted to different photoperiods through the regulation of *FT-like* expression (Chen et al., 2022).

To clarify the relationship between *C. sativa* PEBP members, we searched the cs10/CBDRx genome and compared the PEBP genes identified to those well characterized PEBP genes from the model plant *A. thaliana*, tomato, and the SD plant *Chrysanthemum seticuspe* (Oda et al., 2011). This revealed that there are 12 PEBP family members in *C. sativa*, with four FT-like (*CsFT1* through *CsFT4*), three closely related to *MOTHER OF FT* (*MFT, CsMFT1* through *CsMFT3*), two related to *TERMINAL FLOWER* (*TFL*) and *A. thaliana CENTRORADIALIS* (*ATC*) (*CsATC* and *CsTFL*), as well as three *BROTHER OF FT (BFT*)/*CEN* genes (**Figures 7A, B**, **Supplementary Table S1**). Two of the *CsMFT* clade genes (*CsMFT1*/LOC115711426 and *CsMFT2*/LOC115711470) are almost identical in cs10/CBDRx (**Figure 7B**), with a five-nucleotide insertion/deletion in the 3’ untranslated region, and two synonymous single nucleotide polymorphisms in the coding region. These two genes are also close together on cs10/CBDRx Chr 3 (NC_044372.1) at 92,271,234 - 92,269,219 bp and 92,136,895 - 92,134,894 bp, respectively. To investigate the possibility that these two annotated genes are incorrectly annotated, perhaps because of heterozygosity-induced assembly errors, we examined the genomes of two other cultivars, Finola and Abacus, and could only detect a single *MFT1/2* gene in each case, at the corresponding genomic location. *CsFT1* through *CsFT4* all have a conserved tyrosine at the Y88 position seen in floral promoting-PEBP proteins (**Figure 7B**).

The expression of some PEBP family members in *C. sativa* has been examined in two studies (Chen et al., 2022; Spitzer-Rimon et al., 2022). *CsFT3/LOC115697736, also * (called *FT-like* in (Chen et al., 2022)), exhibits increased expression in the first and second apical leaf pairs following the shift from LD to SD conditions in two wild and two cultivated *C. sativa* strains (Chen et al., 2022). This suggests that the gene may mediate the promotion of flowering in response to a shortening of photoperiod. Six *CsPEBP* genes were differentially expressed in nodes 4 (vegetative), 6 (vegetative) and 7 (reproductive) (Spitzer-Rimon et al., 2022). The three *CsBFT/CEN* genes exhibited reduced expression in node 7, compared to nodes 4 and 6, and *CsMFT3* showed slightly reduced expression. The *CsFT4* gene exhibited increased expression, in node 6, which was unexpected as FT has an amino acid sequence indicative of a floral promoter (Spitzer-Rimon et al., 2022). The expression of *CsTFL* was also reduced in node 6 and node 7, suggesting it may be involved in the maintenance of vegetative function at the shoot apex in vegetative plants. To further clarify the expression of these genes across the whole *C. sativa* plant, we examined their expression in a wide variety of tissues using existing RNASeq datasets and found that the relative expression of *CsMFT1, CsMFT2, and CsMFT3* was greatest in seed, with *CsMFT2* expression reduced in mature Finola flower and Finola root tissues (**Figure 7C**, **Supplementary Table S3**).

### Temperature-dependent pathways in *C. sativa*

The vegetative phase is distinguished by a temperature-dependent basic vegetative phase (BVP) and a daylength-dependent photoperiod induced phase (Lisson et al., 2000). In hemp, a base air temperature of ~1°C and a range of 306 - 636°Cd (thermal time) is required for completion of the BVP (Amaducci et al., 2008; Amaducci et al., 2012). The vegetative stage can also be defined by the number of fully developed leaves (Mediavilla et al., 1998) (**Figure 2A**). While there is little evidence to suggest that *C. sativa* has vernalization requirements, temperature is known to be a factor affecting the length of the juvenile stage, with reduction in temperature increasing the time to floral initiation and flowering (**Supplementary Table S4**) (Lisson et al., 2000; Amaducci et al., 2012; Salentijn et al., 2019).

Temperature contributes to the regulation of flowering time through multiple pathways. In *A*. *thaliana*, the vernalization pathway controls flowering in response to extended cold periods. The vernalization-related gene *VERNALIZATION1* (*VRN1*) codes for a protein that acts to repress the floral repressor TF, FLC (**Figure 6**), subsequently allowing the expression of flowering integrator genes (Michaels and Amasino, 1999; Levy et al., 2002). A *VRN1* ortholog has also been identified in a hemp QTL for full flowering (Petit et al., 2020a) (**Figure 5**). Changes in ambient temperature play a key role in the floral induction of *A. thaliana* under non-inductive SD photoperiods (Balasubramanian et al., 2006; Lee et al., 2007). The type II MADS-box TFs *FLOWERING LOCUS M* (*FLM*) and *SHORT VEGETATIVE PHASE* (*SVP*) assist in regulating ambient temperature-responsive flowering by repressing the expression of florigen genes (Scortecci et al., 2001; Lee et al., 2007). FLM produces multiple splicing variants including FLM-β and FLM-δ, with overexpression of these resulting in late flowering and early flowering, respectively (Pose et al., 2013). At elevated temperatures, ubiquitin-mediated proteasomal degradation reduces SVP while alternative splicing reduces the abundance of FLM-β but increases the abundance FLM-δ (Jin and Ahn, 2021). SVP was also present in our analysis of protein-protein interactions (**Supplementary Figure S2**) and has been shown to inhibit floral transition in the *A. thaliana* autonomous flowering pathway by acting with AGAMOUS-LIKE 24 (AGL24) and AP1 to control floral meristem identity (Gregis et al., 2008).

*FLC* is central to the flowering regulatory network in *A. thaliana* and the control of flowering in response to seasonal cues (Madrid et al., 2020). Floral transition is inhibited by FLC binding directly to genes that encode activators of flowering, to repress their transcription (Michaels and Amasino, 1999; Sheldon et al., 1999). FLC targets *SOC1* (Hepworth et al., 2002; Helliwell et al., 2006; Searle et al., 2006), which encodes a MADS-domain TF that regulates genes involved in floral transition at the shoot apex (Samach et al., 2000; Immink et al., 2012) and assists with floral transition in non-inductive short days (Moon et al., 2003). *SOC1* transcription is activated during vernalization as *FLC* transcription is repressed (Hepworth et al., 2002; Searle et al., 2006; Deng et al., 2011). FLC binds DNA as heterodimers with other members of the MADS-domain TFs family (de Folter et al., 2005; Li et al., 2008; Gu et al., 2013) and, as such, it is important to consider the specificity of MADS-domain complexes including FLC and partner protein availability when examining FLC function and target-specific regulation (Mateos et al., 2015; Madrid et al., 2020).

**Figure 8:**
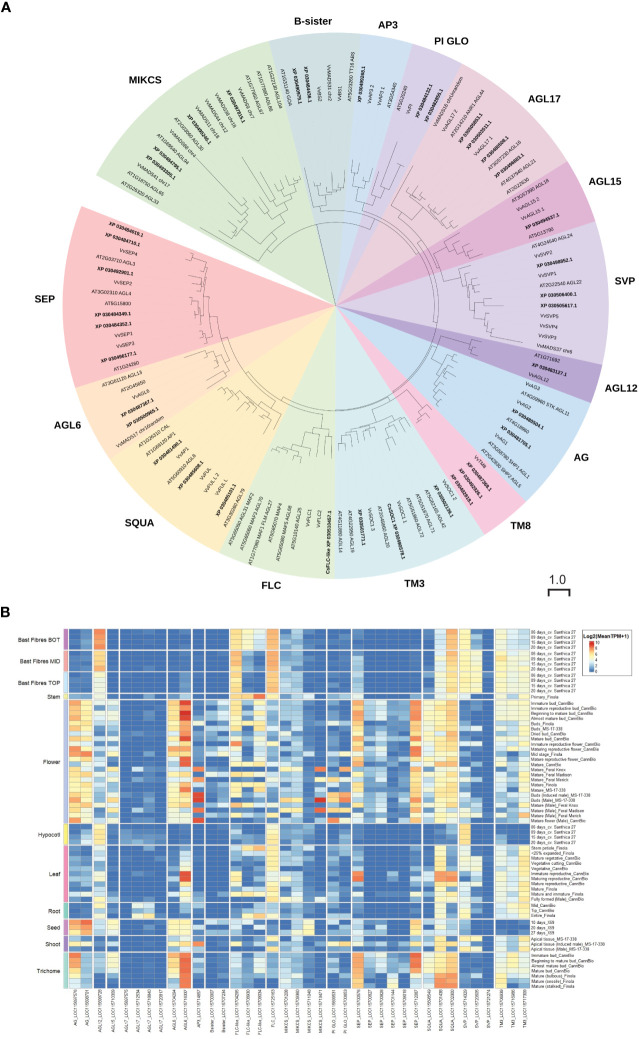
MADS-family proteins in *Cannabis sativa* L. **(A)** Phylogenetic analysis of Type II *C. sativa* MADS family proteins. Proteins were aligned using CLUSTAL in Geneious Prime, and a maximum-likelihood tree was produced using IQ-TREE with JTT+R10 parameters as the best model under AIC and BIC criteria (Minh et al., 2020). The tree was visualized with ITOL (https://itol.embl.de; (Letunic and Bork, 2007)). Numbers indicate percentage bootstrap support following 100 bootstraps (only values above 50 are shown). The scale is the average number of substitutions per site. **(B)** Expression of *CsMADS* family in diverse *C. sativa* tissues. A complete tree of all *C. sativa* MADs proteins is included as **Supplementary Figure S1**. Details of RNASeq data sets are in **Supplementary Table S3**.

There is limited expression data for *SOC1* and *FLC* in *C. sativa* (Chen et al., 2022) (**Figure 6**). To clarify the relationship between *C. sativa* MADS members, we searched the cs10/CBDRx genome and compared the MADS genes identified to those well characterized MADS-box genes from the model plant *A. thaliana*, and grapevine *Vitis vinifera* (**Figure 8**; **Supplementary Figure S1**, **Supplementary Table S2**). This identified one *FLC-like* gene, three *SVP-like* and three *SOC1-like* genes suggesting the involvement of these MADS genes in floral transition in *C. sativa*.

The TF *PHYTOCHROME-INTERACTING FACTOR 4* (*PIF4*) is thought to positively regulate high-temperature-induced flowering by binding to the *FT* promoter region and increasing *FT* transcription (**Figure 6**) (Kumar et al., 2012). *PIF4* transcription is regulated by multiple TFs, with TEOSINTE BRANCHED 1/CYCLOIDEA/PCF 5 (TCP5) thought to positively regulate *PIF4* transcription in response to warm temperatures. Greater ambient temperature increases *PIF4* expression and enhances the accessibility of PIF4, increasing the expression of thermal-responsive genes (Jin and Ahn, 2021). FT binds a membrane phospholipid (phosphatidylglycerol) at low temperatures, restricting mobility. This binding is less preferrable at higher temperatures, allowing FT to travel to the shoot apical meristem and induce flowering. Flowering time is subsequently optimized by the adjustment of florigen (flowering hormone) activity, with cellular membranes sequestering FT by binding the phospholipid, in response to temperature changes (Susila et al., 2021). Similar pathways may exist in *C. sativa*, where one *PIF3* and one *PIF5* homolog are present (**Supplementary Table S1**).

### Autonomous flowering pathways in *C. sativa*

In day-neutral flowering plants, flower induction is primarily regulated by age-dependent, autonomous pathways (Silva et al., 2019). The transition between juvenile and adult developmental phases involves regulation of the levels of microRNAs, *miR156* and *miR172*. *miR156* is highly expressed throughout the juvenile phase and declines prior to flowering. The opposite trend is seen for *miR172*. *miR156* target transcripts of a subset of *SPL* TFs (**Figure 6**) known to promote transition from the juvenile to adult vegetative phases as well as flowering (Wu and Poethig, 2006; Schwarz et al., 2008). In *A. thaliana*, the vegetative phase change is regulated by increased *SPL3* expression due to decreased *miR156* levels (Wu and Poethig, 2006). In maize, the overexpression of *miR156* extends the juvenile phase by 1-2 weeks (Chuck et al., 2007) while the overexpression of *miR172* in *A. thaliana* accelerates flowering (Aukerman and Sakai, 2003; Jung et al., 2007). The abundance of *miR172* is also regulated by photoperiod *via* GI-mediated miRNA processing. GI-regulated *miR172* regulates photoperiodic flowering by inducing *FT* independently of *CO* (Jung et al., 2007). As a result, plants that overproduce *miR172* flower earlier under both long and short days. *miR156* and *miR172* are conserved in *Humulus lupulus*, the closest relative of *C. sativa* (Mishra et al., 2016). Petit et al. (2020a) subjected *C. sativa* microRNAs (Das et al., 2015; Hasan et al., 2016) to a BLASTn (Altschul et al., 1997) search against the genome of *C. sativa* ‘Purple Kush’ assembly (van Bakel et al., 2011) and confirmed the presence of *csa-miR156* and *csa-miR172a*. The conservation of miR156 and miR172a in *C. sativa* suggests they may help determine flowering time alongside 18 SPLs present in *C. sativa* (**Supplementary Table S1**).

Spitzer-Rimon et al. (2022) identified 16 *SPL* genes in *C. sativa*, with expression levels for 13 of these differing significantly between nodes. Expressions patterns could be separated into three groups, the largest of which included *SPLs* upregulated during plant maturation from nodes 4-7 (Spitzer-Rimon et al., 2022). *CsSPL9* exhibited the highest expression levels and may have a key role in regulating the transition between vegetative to reproductive phases. Notably, expression of *CsSPL7* was relatively high in nodes 4 and 6 but sharply downregulated in node 7 (Spitzer-Rimon et al., 2022). *SPL* genes are regulators of the juvenile-to-adult and vegetative-to-reproductive phase transitions in *A. thaliana* (Hyun et al., 2016; Xu et al., 2016; Périlleux et al., 2019), with *SPL9* shown to directly activate expression of *LFY* and *AP1* to promote flowering (Wang et al., 2009; Yamaguchi et al., 2009). In *C. sativa*, nine *SPL* genes are known to be upregulated in the reproductive phase, with *CsAP1* and *CsLFY* upregulated in node 7 alongside *SPL* genes, including *CsSPL9*. (Spitzer-Rimon et al., 2022). Similar mechanisms may be present in the vegetative to reproductive phase transition of *C. sativa*, however, further research is required to better understand the genetic determinants involved in these flowering pathways.

## Conclusions & future prospects

In summary, flowering behavior in *C. sativa* shows a high level of complexity and can vary within and between cultivars, indicating that multiple major and potentially minor effect loci may contribute to these traits. Meta-analysis of available flowering time studies shows 4 co-localized QTL regions. Functional genomic analyses focusing on these genetic intervals and other loci identified in this review will be essential to improve our understanding of the genetic basis underlying flowering behavior in *C. sativa*.

Recently, the efficacy of virus-induced gene silencing (VIGS) and virus-aided gene expression (VAGE) has been demonstrated in *C. sativa* (Schachtsiek et al., 2019; Alter et al., 2022), which offers opportunities to test the function of the putative flowering time gene candidates (Spitzer-Rimon et al., 2022; Toth et al., 2022). *Autoflower1* genes (including *RAP2-7*, *UPF* and *Early1*) are obvious targets for such analysis using transient gene-expression modification systems, with even transient reductions in gene expression likely to result in altered flowering times in inductive or non-inductive photoperiods. The prospect for functional analysis of flowering time by stable transformation incorporating overexpression or gene editing systems appears more elusive, with few reports of viable or reproducible transformation protocols yielding stably transformed plants (Galán-Ávila et al., 2021; Zhang et al., 2021). The recent development of molecular markers tightly linked to the *Autoflowering* trait on chromosome 1 offers great promise in *C. sativa* breeding programs. In the future, tightly controlled studies of *C. sativa* populations are likely to identify further markers.

While much of the work on flowering time regulation is protein-centric, plant metabolites also play a key role in regulating flowering. Metabolomic analysis could be used to identify metabolites with greater abundance in early or late flowering *C. sativa* genotypes, for use as potential biomarkers in breeding trials (Arkhimandritova et al., 2020). Gene expression profiling has potential to reveal the mode of action of small molecules in *C. sativa*, such as 4-dibromo-7-azaindole (B-AZ) which has been shown to lengthen the circadian period and inhibit the Casein Kinase 1 family (CK1) in *A. thaliana* (Ono et al., 2019). A chemical genomics screening platform has also been successfully used to discover compounds that can induce flowering in *A. thaliana* and a similar approach could be developed in *C. sativa* (Fiers et al., 2017).

Given the phenotypic plasticity in *C. sativa*, epigenetic regulation may influence flowering behavior. The DNA demethylating agent 5-azacytidine induces non-vernalized *A. thaliana* plants to flower significantly earlier than untreated controls (Burn et al., 1993). Late-flowering mutants insensitive to vernalization do not respond to 5-azacytidine treatment, suggesting that DNA methylation prevents early flowering (Burn et al., 1993). Temperature-sensitive lipid binding has also been demonstrated to assist in the timing of flowering with favorable ambient temperatures (Susila et al., 2021) and histone deacetylase-mediated transcriptional repression may result in changes to flowering behavior, with antisense inhibition of the expression of histone deacetylase *HDA19* (or *AtHD1*) resulting in delayed flowering in *A. thaliana* (Wu et al., 2000). These and other emerging technologies could be employed to regulate *C. sativa* flowering with improved precision and accuracy, thereby offering opportunities to optimize commercial cultivation and improve yields of valuable feedstocks used for industrial and medicinal end-uses.

## Data availability statement

The datasets presented in this study can be found in online repositories. The names of the repository/repositories and accession number(s) can be found in the article/**Supplementary Material**.

## Author contributions

MW, KJ and AG provided substantial contributions to conception and design of the research project and performed detailed review and revision of the manuscript. LS wrote the manuscript, conducted the DIAMOND and BLASTn analysis and generated the protein-protein interaction network. NR conducted the gene expression and MADS phylogenetic analyses. AG conducted comparative genomic analyses. All authors contributed to the article and approved the submitted version.

## Supplementary material

Supplementary Figure 1 MADS protein phylogeny.

Supplementary Figure 2 Analysis of flowering time protein-protein interactions.

Supplementary Table 1
*C. sativa* flowering genes.

Supplementary Table 2 PEBP and MADS proteins.

Supplementary Table 3 RNA Seq data accessions.

Supplementary Table 4 Summary of flowering time measurement schemes in female C. sativa plants.
