# Supplementary figures and images for: Comparative genomics of flowering behavior in Cannabis sativa
Source: Front Plant Sci. 2023 Jul 27;14:1227898. doi: 10.3389/fpls.2023.1227898 (PMC10421669; doi:10.3389/fpls.2023.1227898)

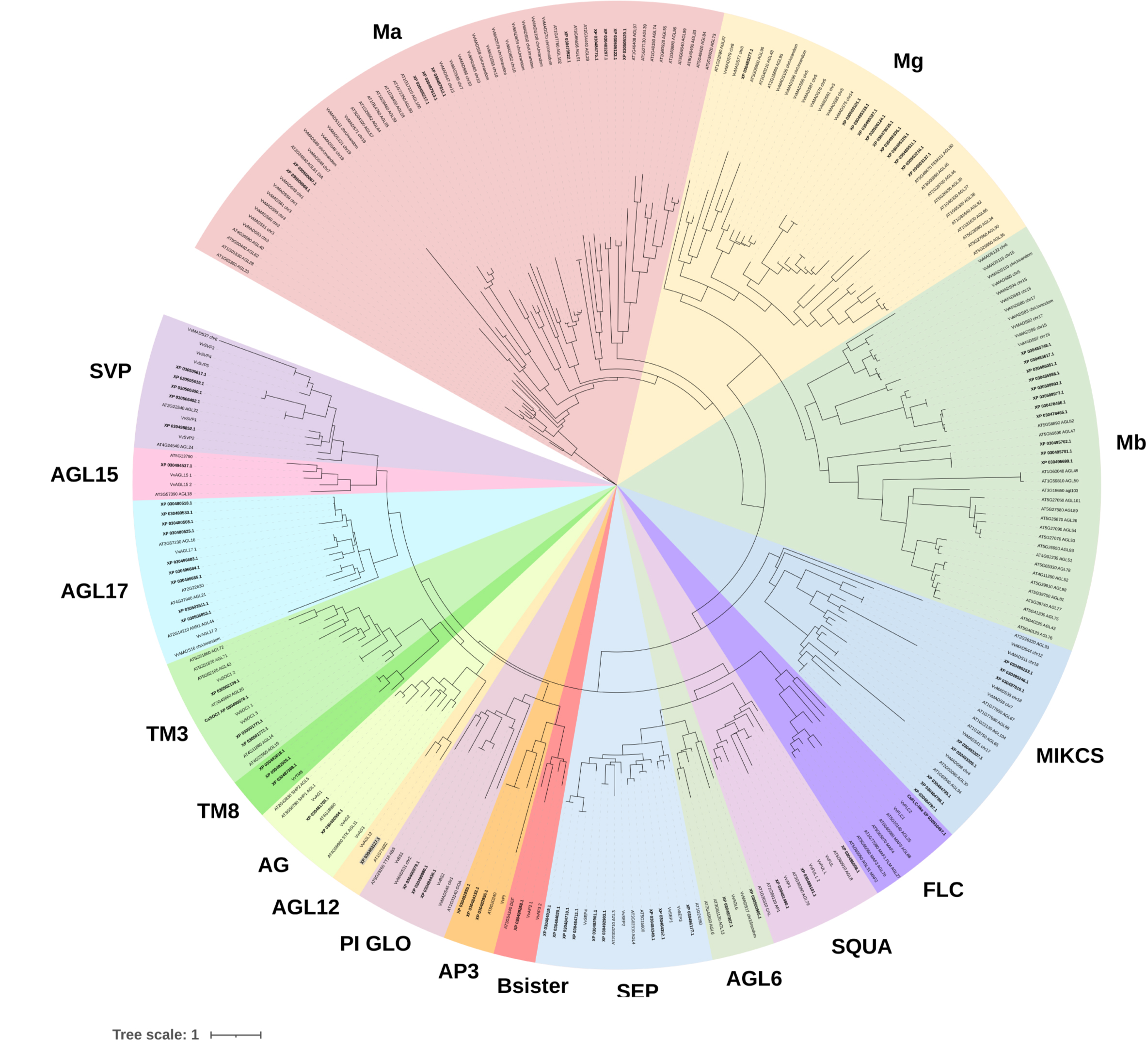

Supplement: Supplementary Figure 1 — MADS protein phylogeny. [file Image_1.jpg]

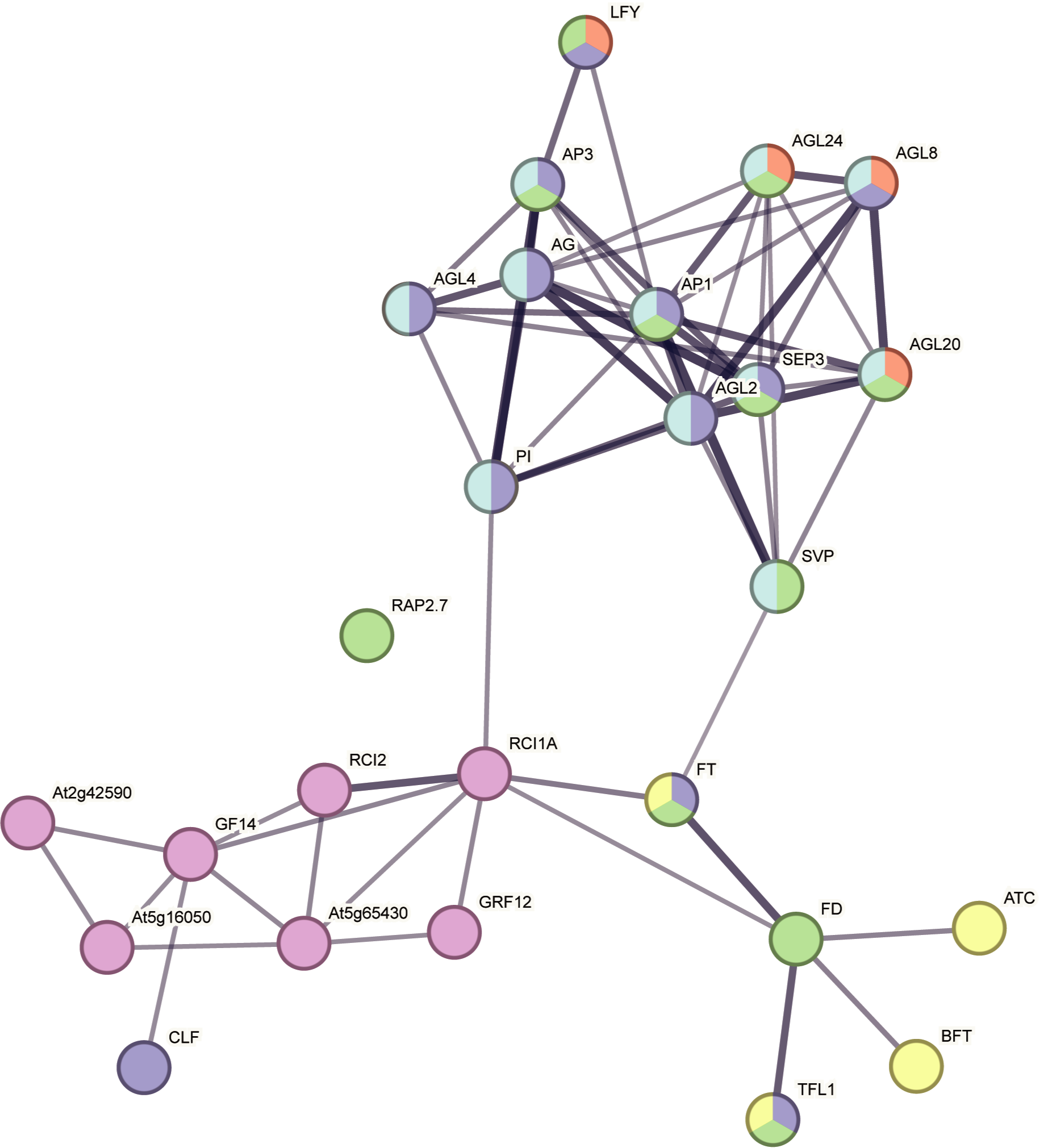

Supplement: Supplementary Figure 2 — Analysis of flowering time protein-protein interactions. [file Image_2.tif]
