# Supplementary material for: Comparative genomics of flowering behavior in Cannabis sativa
Source: Front Plant Sci. 2023 Jul 27;14:1227898. doi: 10.3389/fpls.2023.1227898 (PMC10421669; doi:10.3389/fpls.2023.1227898)
Supplement: Supplementary Table 4 — Summary of flowering time measurement schemes in female C. sativa plants. [file Table_4.docx]

|  |  |  |  | **Proposed main events of florogenesis in female C. sativa plants** | | | | | **Seed Maturation** | | |
| --- | --- | --- | --- | --- | --- | --- | --- | --- | --- | --- | --- |
| **Research paper** | **Late vegetative phase** | **Floral initiation** | **Bract formation** | **Induction of solitary flowers** | **Higher order branching** | **Formation of flower clusters at shoot apices consisting of two or more pairs of stigmata** | **Terminal flower differentiation at the apical meristem** | | **Beginning of seed maturity** | **Seed Maturity** | **End of seed maturity** |
| Mediavilla et al., 1998 | 2000 GV point | 2001 Flower primordia | 2200  First perigonal bracts | 2201  Beginning of flowering: Styles of first female flowers |  | 2202  Flowering: 50% of bracts formed | 2102  Pollen is released | 2103  95% of staminate flowers opened or withered | 2203  First seeds hard | 2204  50% of seeds hard | 2205  95% of seeds hard or shattered |
| Raghavan, 2000 | Plants considered vegetative | Floral initiation | Floral organisation & maturation | | | | Anthesis | |  |  |  |
| Lisson et al., 2000 | Plants considered vegetative | Floral initiation: 50%+ harvested plants have 1+ floral primordia | Flower development phase | Appearance of first flower |  | Flower formation: 50%+ plants have stigmatic female flowers | Harvest maturity (male anthesis): 50% of male plants shedding pollen |  |  |  |  |
| Spitzer-Rimon et al., 2019 |  |  |  | Development of two bracts located on each side of the leaf petiole base, each subtending a solitary flower | Stigmata form at top of main shoot. Apical meristems of main shoot and lateral branches remain indeterminate and continued producing phytomers | Inflorescence flowering: at least three pairs of stigmata are visible at the top of the apical shoot | Full-flowering stage, main inflorescences noticeable on the apical part of the main, second-, and third-order branches | |  |  |  |
| Braich et al., 2019  (Featured in Ren et al., 2021) |  | Pre-flowers: shoot tips without visible stigma | | | Early stage: flowers with visible stigma | | Mid-stage: flowers with non-withered stigmas and visible trichomes | |  |  |  |
| Petit et al., 2020 |  |  |  | Beginning of flowering: the accumulated thermal time over a period with a base temperature of 1°C relative to the day of emergence |  |  | Full flowering: the accumulated thermal time over a period with a base temperature of 1°C relative to the day of emergence | |  |  |  |
| Woods et al., 2021 |  |  |  |  |  |  |  |  | Plant maturity/Seed maturity: bracts being to dehisce, darkening of seed coat visible (Campbell et al., 2019) |  |  |
| Chen et al. 2021 |  |  | Flowering time: Flower buds visible at the top of male plants. Staminate flowers usually appear about two weeks before the styles of female plants (Clarke 1997). |  |  |  |  |  |  |  |  |
| Stack et al., 2021/Toth et al., 2022 |  |  | Male flowering: length of internodes at plant apex shorten and male buds are clearly visible at the growing tip | Sparse, solitary flowers developing in the axils of the leaves (Spitzer-Rimon et al., 2019) |  |  | Terminal flowering: clusters of female flowers at shoot apices | |  |  |  |
| Kurtz et al., 2023 |  |  |  |  |  | Initiation of terminal flowering: a minimum of three pistils bearing stigmas were visible at the shoot tips (Spitzer-Rimon et al., 2019) | Peak flowering: terminal colas appear to reach their maximum size and trichomes begin to change from clear to translucent white | |  |  |  |
| Peterswald et al., 2023 |  | Prescence of pistils |  |  |  |  |  | |  |  |  |
| Dowling et al., 2023 |  |  | Male flowering: individual male flowers larger than 2 mm visible | Female flowering: at least one pair of stigmata visible |  |  |  | |  |  |  |
